# Maternal and obstetric outcomes following the transfer of embryos warmed with fatty acid-supplemented solutions

**DOI:** 10.1186/s12884-024-06546-4

**Published:** 2024-05-04

**Authors:** Kenji Ezoe, Sachie Onogi, Ayano Sawado, Ayumi Amagai, Keiichi Kato

**Affiliations:** Kato Ladies Clinic, 7-20-3 Nishishinjyuku, Shinjyuku-ku, Tokyo, 160-0023 Japan

**Keywords:** Cleavage-stage embryo transfer, Fatty acid, Live birth, Perinatal outcomes, Vitrification

## Abstract

**Background:**

Vitrification procedures decrease intracytoplasmic lipid content and impair developmental competence. Adding fatty acids (FAs) to the warming solution has been shown to recover the lipid content of the cytoplasm and improve developmental competence and pregnancy outcomes. However, the influence of the FA supplementation on live birth rates after embryo transfers and perinatal outcomes remains unknown. In the present study, we examined the influence of FA-supplemented warming solutions on live birth rates, pregnancy complications, and neonatal outcomes after single vitrified-warmed cleavage-stage embryo transfers (SVCTs).

**Methods:**

The clinical records of 701 treatment cycles in 701 women who underwent SVCTs were retrospectively analyzed. Vitrified embryos were warmed using solutions (from April 2022 to June 2022, control group) or FA-supplemented solutions (from July 2022 to September 2022, FA group). The live birth rate, pregnancy complications, and perinatal outcomes were compared between the control and FA groups.

**Results:**

The live birth rate per transfer was significantly higher in the FA group than in the control group. Multivariate logistic regression analysis further demonstrated a higher probability of live births in the FA group than in the control group. Miscarriage rates, the incidence and types of pregnancy complications, the cesarean section rate, gestational age, incidence of preterm delivery, birth length and weight, incidence of low birth weight, infant sex, and incidence of birth defects were all comparable between the control and FA groups. Multivariate logistic regression analysis further demonstrated no adverse effects of FA-supplemented warming solutions.

**Conclusions:**

FA-supplemented warming solutions improved live birth rates after SVCTs without exerting any adverse effects on maternal and obstetric outcomes. Therefore, FA-supplemented solutions can be considered safe and effective for improving clinical outcomes and reducing patient burden.

**Supplementary Information:**

The online version contains supplementary material available at 10.1186/s12884-024-06546-4.

## Background

The cycle number of frozen embryo transfers (FETs) has recently increased owing to improvements in vitrification techniques and increased application of the freeze-all strategy [1–3]. However, previous studies have indicated adverse effects of vitrification on subsequent development after warming [4–6]. The altered characteristics of cytoplasmic organelles and increased abnormalities in chromosomal segregation reduce the developmental competence of vitrified-warmed oocytes and embryos [7–9]. Furthermore, our recent study revealed that vitrification procedures decrease intracytoplasmic lipid droplets and impair developmental competence [10]. However, lipid droplets in the cytoplasm could be recovered and developmental competence was improved by the addition of long-chain fatty acids (FAs) to the warming solutions (Supplementary Table 1). Moreover, we demonstrated that the rates of implantation, clinical pregnancy, and ongoing pregnancy after single vitrified-warmed cleavage-stage embryo transfer (SVCT) significantly increased after warming with FA-supplemented solutions. However, whether FA supplementation improves live birth rates following SVCTs is currently unknown. In addition, previous studies have indicated that pregnancies resulting from in vitro fertilization are associated with a higher risk of adverse maternal and perinatal outcomes such as hypertensive disorders of pregnancy, low birth weight, small for gestational age (SGA) and large for gestational age (LGA), preeclampsia, and placental anomalies [11–15]. Therefore, one future focus in reproductive medicine should be to assess whether assisted reproduction technology (ART) is associated with increased risks of these complications. From this perspective, the effectiveness and safety of FA-supplemented solutions should be evaluated. Accordingly, in the present study, we retrospectively analyzed the live birth rates, maternal outcomes, and perinatal outcomes after the transfer of cleavage-stage embryos warmed with FA-supplemented solutions.

## Methods

### Ethical approval

This retrospective cohort study was approved by the Institutional Review Board of our center (approval number: 21–12). Written informed consent for the retrospective use of data was obtained from all patients.

### Study patients

The clinical records of 730 treatment cycles in 730 women who underwent SVCTs in natural cycles between April 2022 and September 2022 were reviewed. Patients with recurrent implantation failure (four or more unsuccessful FETs [16], *n* = 25), those who underwent procedures involving artificial oocyte activation (*n* = 3), and those with surgically retrieved sperm (*n* = 1) were excluded. Each case in this study involved only one cycle per patient.

### Embryo warming

From April 2022 to June 2022, vitrified embryos were warmed using solutions that were not supplemented with FAs (VT506; Kitazato Corporation, Shizuoka, Japan; control group). From July 2022 to September 2022, vitrified embryos were warmed in FA-supplemented solutions (VT526; Kitazato Corporation, FA group; Supplementary Table 1). The warming procedures were performed using the Cryotop method [10, 17]. Briefly, the Cryotop was placed in a solution of 1.0 M sucrose at 37 °C for 1 min. The blastocysts were then removed after warming and transferred to a diluent sucrose solution (0.5 M) at 26–28 °C. After 3 min, the cells were transferred to a washing solution without sucrose and washed for 1 min.

### Embryo transfer

SVCT was performed under vaginal ultrasound guidance using a specially designed soft silicone inner catheter (Kitazato Corporation), as previously described [18]. A single embryo was placed in a minimal volume in the upper part of the uterine cavity on day 2 after ovulation in a natural cycle. Dydrogesterone (30 mg/day; Mylan EPD G.K.) was orally administered during the early luteal phase after SVCT.

Live birth was defined as delivery at ≥ 22 weeks of gestation. Miscarriage was defined as the absence of live birth after confirmation of a gestational sac [19]. Maternal and obstetric outcomes were obtained from a self-reported questionnaire completed by each patient after the infant’s 1-month examination. All pregnant women were invited to respond to the questionnaire at 9 weeks of gestation, in the second trimester, and after delivery [20]. Maternal and obstetric outcomes included hypertensive disorders of pregnancy, gestational diabetes mellitus, hemolysis-elevated liver enzymes, low platelet count syndrome, low-lying placenta, placenta previa, placental abruption, and cesarean section. A low-lying placenta was determined in cases where the edge of the placenta was < 20 mm from the cervix, but not overlying it. When the placenta completely covered the cervix, it was classified as placenta previa [21]. Neonatal outcomes included gestational age, birth length and weight, SGA, and LGA. SGA and LGA were defined as birth weights below the 10th percentile and above the 90th percentile, respectively, according to the Japanese National Reference for neonates [22]. Congenital anomalies were classified using the Q codes of the International Statistical Classification of Diseases and Related Health Problems, 10th Revision, by reformatting the answers provided by parents in the questionnaires [23]. Major congenital anomalies were classified according to the European Surveillance of Congenital Anomalies guidelines [24].

### Statistical analysis

Statistical analyses were performed using JMP software (SAS, Cary, NC, USA). Data proportions were analyzed using the chi-square and Fisher’s exact tests. Continuous parameters were compared using the Mann–Whitney U test. Univariate regression analysis was used to identify potential confounders associated with the outcomes. The likelihood ratio test for the significance of the regression coefficient was used, and variables with *P* < 0.10 were designated as confounders (Supplementary Table 2). Multivariate logistic regression analysis was performed to adjust for bias and to verify statistical significance. Adjusted odds ratios are reported with 95% confidence intervals for each group. *P* < 0.05 was considered significant.

## Results

### Live births after SVCTs

The patient and cycle characteristics were comparable between the control and FA groups (Table 1). The delivery rate was significantly higher in the FA group than in the control group (Table 2). As the live birth rate per delivery was 100% in both groups, the live birth rate per transfer was significantly higher in the FA group than in the control group. Miscarriage rates were comparable between the two groups. Multivariate logistic regression analysis also demonstrated a higher probability of live births in the FA group than in the control group (Table 3).

**Table 1:** Characteristics of the study cohort

	Control group	Fatty acid group	*P* value
Number of couples, n	340	361	
Embryo transfer cycles, n	340	361	
Maternal age, mean ± SEM	37.1 ± 0.2	36.9 ± 0.2	0.471
Paternal age, mean ± SEM	39.6 ± 0.3	39.5 ± 0.3	0.898
Body mass index, mean ± SEM	21.2 ± 0.2	20.8 ± 0.1	0.094
Smoking, n (%)	0 (0)	0 (0)	–
Previous embryo transfer, mean ± SEM	0.7 ± 0.0	0.6 ± 0.0	0.191
Previous delivery, n (%)	107 (31.5)	101 (28.0)	0.312
Previous miscarriage, n (%)	80 (23.5)	92 (25.5)	0.548
Cause of infertility			0.087
Ovulation, n (%)	28 (8.2)	38 (10.5)	
Tubal factor, n (%)	3 (0.9)	8 (2.2)	
Endometriosis, n (%)	12 (3.5)	15 (4.2)	
Endometrial factor, n (%)	27 (7.9)	17 (4.7)	
Male factor, n (%)	102 (30.0)	95 (26.3)	
Combined, n (%)	62 (18.2)	51 (14.1)	
Unexplained, n (%)	106 (31.2)	137 (38.0)	
Insemination			0.897
Conventional in vitro fertilization	134 (39.4)	144 (39.9)	
Intracytoplasmic sperm injection	206 (60.6)	217 (60.1)	
No. of blastomeres of the transferred embryos, mean ± SEM	5.4 ± 0.1	5.4 ± 0.1	0.658
Morphological grade of the transferred embryos, n (%)			0.257
Grade 1	54 (15.9)	44 (12.2)	
Grade 2	107 (31.5)	108 (29.9)	
Grade 3	179 (52.7)	209 (57.9)	
Endometrial thickness on the day of ET, mean ± SEM	10.1 ± 0.1	10.1 ± 0.1	0.551

Values are presented as mean ± SEM or n (%). SEM, standard error of the mean; ET, embryo transfer. Each case in this study involved only one cycle per patient

**Table 2:** Pregnancy outcomes after single vitrified-warmed cleavage stage embryo transfers

	Control group	Fatty acid group	*P* value
Embryo transfer cycles, n	340	361	
Delivery, n (%)	64 (18.9)	91 (25.4)	0.040
Live birth/delivery, n (%)	64 (100)	91 (100)	–
Live birth/transfer, n (%)	64 (18.9)	91 (25.4)	0.040
Miscarriage, n (%)	23 (26.4)	30 (25.0)	0.815

Values are presented as n (%)

**Table 3:** Multivariate logistic regression analysis for live birth

	Adjusted odds ratio*	95% confidential interval	*P* value	Area under the curve
Maternal age	0.93	0.87–0.98	0.005	0.634
Paternal age	0.98	0.93–1.02	0.306	
Warming solution				
Control group	Reference	–	–	
Fatty acid group	1.45	1.00–2.09	0.048	

*Confounders: maternal age, paternal age

Patients were stratified by median maternal age for further analysis (37 years; Supplementary Table 3). This subgroup analysis showed that the live birth rate per transfer was significantly higher in the FA group than in the control group in women younger than 37 years of age. Multivariate logistic regression analysis also demonstrated a higher probability of live births in the FA group than in the control group in the young cohort. However, the live birth rate per transfer was comparable between the two groups for women aged 37 years or older. The incidence of miscarriage was comparable between the groups in both the young and advanced-age cohorts.

### Maternal and obstetric outcomes after SVCTs

The incidence and types of pregnancy complications were comparable between the control and FA groups (Table 4). Furthermore, cesarean section rates were similar between the groups (Table 4). The average gestational age and incidence of preterm delivery were comparable between the groups. Birth length and weight, incidence of low birth weight, incidence of SGA and LGA, and infant sex were comparable between the groups. The incidence of birth defects was also similar between the groups. Multivariate logistic regression analysis revealed a similar trend (Table 5).

**Table 4:** Pregnancy complications and neonatal outcomes after single vitrified-warmed cleavage stage embryo transfers

	Control group	Fatty acid group	*P* value
Deliveries, n	64	91	
Pregnancy complications, n (%)	3 (4.7)	4 (4.4)	0.931
Hypertensive disorders of pregnancy, n (%)	1 (1.6)	1 (1.1)	0.801
Gestational diabetes mellitus, n (%)	0 (0)	1 (1.1)	0.400
HELLP syndrome, n (%)	1 (1.6)	0 (0)	0.232
Low-lying placenta, n (%)	1 (1.6)	0 (0)	0.232
Placenta previa, n (%)	0 (0)	1 (1.1)	0.400
Placental abruption, n (%)	0 (0)	1 (1.1)	0.400
Follow-up data, n	63	89	
Caesarean section rate, n (%)	14 (22.2)	26 (29.2)	0.335
Gestational age, weeks, mean ± SEM	39.3 ± 0.2	39.3 ± 0.1	0.928
Preterm delivery (< 37 weeks)	2 (3.2)	3 (3.4)	0.947
Birth length, cm, mean ± SEM	49.2 ± 0.3	49.4 ± 0.2	0.668
Birth weight, g, mean ± SEM	3,055 ± 51	3,054 ± 44	0.990
Low birth weight (< 2,500 g)	3 (4.8)	6 (6.7)	0.610
Small for gestational age	2 (3.1)	5 (5.5)	0.484
Large for gestational age	10 (15.6)	19 (20.9)	0.409
Infant sex			0.067
Male, n (%)	27 (42.2)	52 (57.1)	
Female, n (%)	37 (57.8)	39 (42.9)	
Birth defect, n (%)	2 (3.1)	0 (0)	0.090

HELLP; hemolysis, elevated liver enzymes, low platelet count. Values are presented as mean ± SEM or n (%). SEM, standard error of the mean

**Table 5:** Multivariate logistic regression analysis of perinatal outcomes

Adverse neonatal outcomes	Adjusted odds ratio (95% confidence intervals) *^1^	95% confidence intervals	*P* value
Pregnancy complication	1.32*^2^	0.27–6.49	0.373
Caesarean section	1.61*^3^	0.74–3.49	0.228
Preterm delivery (< 37 weeks)	1.46*^2^	0.22–9.53	0.687
Low birth weight (< 2,500 g)	1.54*^4^	0.35–6.66	0.558
Small for gestational age	1.80*^3^	0.33–9.59	0.490
Large for gestational age	1.42*^3^	0.61–3.31	0.413

*^1^ Reference: control group. *^2^ Confounder: endometrial thickness. *^3^ Confounder: body mass index. *^4^ Confounders: maternal age and paternal age

## Discussion

To the best of our knowledge, this is the first report to show a significant improvement in live birth rates after the transfer of cleavage-stage embryos warmed with FA-supplemented solutions. Furthermore, we confirmed that these solutions had no adverse effects on maternal or obstetric outcomes.

In the present study, we first examined the effects of FA-supplemented warming solutions on live birth rates after SVCTs. The live birth rate per transfer was significantly higher in the FA group than in the control group. This result validates our previous finding that FA-supplemented solutions improved the ongoing pregnancy rate [17]. Furthermore, the miscarriage rate was unchanged when FA-supplemented solutions were used. The enzyme-mediated β-oxidation of FAs is a crucial metabolic process in mitochondria and is essential for early embryo development [25, 26]. Through β-oxidation, FAs are catabolized to acetyl-CoA, which leads to adenosine triphosphate (ATP) production via the mitochondrial electron transport chain [26–28]. Furthermore, the FA-supplemented solutions stimulate the blastocyst formation rate and improve embryo quality [10, 17], suggesting that β-oxidation-induced developmental improvements would lead to an increased chance to achieve live birth after warming of cleavage stage embryos with FA-supplemented solutions. Furthermore, we performed a subgroup analysis in which patients were stratified according to the median maternal age. The probability of live births significantly improved using FA-supplemented solutions only when the patients were young (< 37 years of age). A recent study indicated that mitochondrial function in embryos declines as maternal age increases [29]. Therefore, we speculated that even if the embryos from the advanced age patients possess sufficient metabolic substances, such as FAs, they could not produce sufficient ATP through β-oxidation. Further molecular studies are required to compare the activity of β-oxidation after warming with the FA-supplemented solutions between young females and those of advanced age.

Our data also demonstrate that the incidence of pregnancy-related complications was not affected by the FA-supplemented solutions. Furthermore, the perinatal outcomes, including infant health, were comparable between the control and FA groups. Previous studies have reported that vitrification increases the risk of pregnancy-related complications [11, 12]. However, most FETs in these studies were performed during hormone replacement cycles. In a previous study, we found that endometrial preparation during the hormone replacement cycle, rather than vitrification procedures, adversely affected pregnancy complications compared to endometrial preparation during the natural cycle [30]. The FA-supplemented solutions did not have adverse effects on maternal or obstetric outcomes. Therefore, we determined the efficacy and safety of the solutions in the present study.

One strength of this study is that we examined maternal and obstetric data obtained from a single center, and the culture conditions and SVCT procedures were uniform. Moreover, we included the characteristics of patients, cycles, and embryos as confounders that were significantly associated with the live birth rate and incidence of adverse maternal and perinatal outcomes after SVCTs in multivariate logistic regression analysis. Naturally, this study had certain limitations. For example, the single-center retrospective design limits the strength of the study, necessitating further multicenter studies to ascertain the generalizability of these findings to other clinics with different protocols and/or patient demographics. Lipid droplets are regulated at the homeostatic level to participate in cellular membrane formation, and they play a versatile role as an energy source [31–33]. The lipid droplets in early cleavage-stage embryos are small and are dispersed throughout the cytoplasm or may be partially clustered, whereas larger lipid droplets are formed after compaction, which allows for increased contact area with other organelles, such as mitochondria, thereby facilitating lipid exchange, replenishment, and FA β-oxidation [32, 34]. Furthermore, it has been recently reported that the biosynthesis of unsaturated FAs is activated at the blastocyst stage in mice and humans, and this activation is necessary for the formation of the apical and basal domains to establish polarity toward blastocyst development [31, 35]. This suggests that the detrimental effects of vitrification on intracellular lipid droplets vary by developmental stage. Further molecular studies, such as a lipidome analysis, are required to reveal which FAs decrease after vitrification at each stage. Although the effectiveness of FA-supplemented warming solutions for frozen blastocyst transfers remains unclear, it is hypothesized that warming of blastocysts using FA-supplemented solutions may positively affect blastocyst viability and competence for implantation since the FA-supplemented solutions contain several unsaturated FAs, such as oleic acid, that are believed to improve embryo membrane fluidity and subsequent development [35]. To test this hypothesis, further experimental and clinical studies are required. Another limitation of our study is that the number of perinatal cycles was low. Therefore, larger cohort studies are required to substantiate our findings. Finally, although the same protocols were used for ovarian stimulation, oocyte retrieval, embryo culture, and embryo transfer, the control and FA group data were collected at different time periods.

## Conclusion

The FA-supplemented warming solutions improved the live birth rates after SVCTs, particularly in young patients. Furthermore, infant health and the incidence of pregnancy complications were not affected by the FA-supplemented solutions. As such, FA-supplemented solutions can be considered safe and effective at improving clinical outcomes and reducing patient burden.

### Electronic supplementary material

Below is the link to the electronic supplementary material.

Supplementary Material 1

## Data Availability

The datasets used and/or analyzed during the current study are available from the corresponding author on reasonable request.
