# Supplementary material for: Maternal and obstetric outcomes following the transfer of embryos warmed with fatty acid-supplemented solutions
Source: BMC Pregnancy Childbirth. 2024 May 4;24:343. doi: 10.1186/s12884-024-06546-4 (PMC11069166; doi:10.1186/s12884-024-06546-4)
Supplement: Supplementary file 1 — Supplementary Material 1 [file 12884_2024_6546_MOESM1_ESM.docx]

Supplementary Table 1. Fatty acid composition in warming solutions

| Type of fatty acids | Length of fatty acids | Component of fatty acids | Concentration (nM) |
| --- | --- | --- | --- |
| Unsaturated fatty acid | Long-chain | Arachidonic acid | 65.7 |
|  | Long-chain | Linoleic acid | 356.6 |
|  | Long-chain | Linolenic acid | 359.2 |
|  | Long-chain | Oleic acid | 354.0 |
|  | Long-chain | Palmitoleic acid | 393.0 |
| Saturated fatty acid | Long-chain | Myristic acid | 437.9 |
|  | Long-chain | Palmitic acid | 390.0 |
|  | Long-chain | Stearic acid | 351.5 |

Supplementary Table 2. *P* values of univariate logistic regression analysis between the confounder and outcomes

|  | Maternal age | Paternal age | Body mass index | Previous embryo transfers | Previous delivery | Cause of infertility | Endometrial thickness | Morphological grade | Infant sex |
| --- | --- | --- | --- | --- | --- | --- | --- | --- | --- |
| Live birth | *P* < 0.001 | *P* < 0.001 | *P* = 0.327 | *P* = 0.382 | *P* = 0.229 | *P* = 0.158 | *P* = 0.124 | *P* = 0.199 |  |
| Miscarriage | *P* = 0.099 | *P* = 0.752 | *P* = 0.320 | *P* = 0.894 | *P* = 0.822 | *P* = 0.362 | *P* = 0.126 | *P* = 0.375 |  |
| Pregnancy complications | *P* = 0.936 | *P* = 0.434 | *P* = 0.838 | *P* = 0.284 | *P* = 0.775 | *P* = 0.318 | *P* = 0.029 | *P* = 0.478 | – |
| Caesarean section | *P* = 0.549 | *P* = 0.495 | *P* = 0.091 | *P* = 0.150 | *P* = 0.103 | *P* = 0.156 | *P* = 0.811 | *P* = 0.682 | *P* = 0.810 |
| Preterm delivery | *P* = 0.762 | *P* = 0.469 | *P* = 0.859 | *P* = 0.293 | *P* = 0.190 | *P* = 0.811 | *P* = 0.089 | *P* = 0.730 | *P* = 0.128 |
| Low birth weight | *P* = 0.026 | *P* = 0.025 | *P* = 0.738 | *P* = 0.154 | *P* = 0. 482 | *P* = 0.709 | *P* = 0.150 | *P* = 0.625 | *P* = 0.150 |
| Small for gestational age | *P* = 0.360 | *P* = 0.231 | *P* = 0.093 | *P* = 0.752 | *P* = 0.775 | *P* = 0.675 | *P* = 0.810 | *P* = 0.507 | *P* = 0.810 |
| Large for gestational age | *P* = 0.927 | *P* = 0.840 | *P* = 0.090 | *P* = 0.620 | *P* = 0.322 | *P* = 0.699 | *P* = 0.448 | *P* = 0.338 | *P* = 0.448 |

Supplementary Table 3. Pregnancy outcomes following single vitrified-warmed cleavage stage embryo transfers, stratified by maternal age

|  | Control | Fatty acid | *P* value | Adjusted odds ratio*^1^ | 95% CI | *P* value |
| --- | --- | --- | --- | --- | --- | --- |
| **Maternal age: < 37 y** |  |  |  |  |  |  |
| Embryo transfer cycles, n | 144 | 157 |  |  |  |  |
| Live birth/transfer, n (%) | 31 (21.5) | 54 (34.4) | 0.013 | 1.91*^2^ | 1.14–3.21 | 0.014 |
| Miscarriage, n (%) | 10 (24.4) | 12 (18.5) | 0.464 | 0.77*^3^ | 0.29–2.071 | 0.598 |
| **Maternal age: ≥ 37 y** |  |  |  |  |  |  |
| Embryo transfer cycles, n | 195 | 202 |  |  |  |  |
| Live birth/transfer, n (%) | 33 (16.9) | 37 (18.3) | 0.716 | 1.05*^2^ | 0.62–1.78 | 0.864 |
| Miscarriage, n (%) | 13 (28.3) | 18 (32.7) | 0.628 | 1.23*^3^ | 0.52–2.96 | 0.646 |

*^1^ Reference: control group. *^2^ Confounders: maternal age, paternal age. *^3^ Confounder: maternal age. CI, confidence interval; y, years
